# Saccharine

**Published:** 1889-07

**Authors:** 


Saccharine.—Saccharine is regarded by a French writer (*London Lancet*) as a valuable antiseptic. A strength of 1 to 500, as an addition to mucilaginous and other solutions, prevents the formation of low organisms. Thus a valuable, inexpensive dentrifice may be prepared by simply dissolving saccharine in water, to the proportion of six per cent. A teaspoonful of this in a half pint of water forms an admirable antiseptic mouth wash. In cases of malignant or other diseases of the stomach, requiring the washing out of that organ, a solution of saccharine of the strength of two per cent. will be found suitable.

